# Families’ experiences of end-of-life care in an acute private hospital: A qualitative study

**DOI:** 10.1017/S1478951525000045

**Published:** 2025-02-28

**Authors:** Rosemary Saunders, Susan Alexander, Julie Andrew, Anne Wilkinson, Karen Gullick, Ashwini Davray, Manonita Ghosh, Karla Seaman, Michelle Gay

**Affiliations:** 1 Centre for Research in Aged Care, School of Nursing & Midwifery, Edith Cowan University, Joondalup, WA, Australia; 2 Social Ageing Future Lab, School of Arts and Humanities, Edith Cowan University, Joondalup, WA Australia; 3 School of Arts and Humanities, Edith Cowan University, Joondalup, WA, Australia; 4 Centre for Health Systems and Safety Research, Australian Institute of Health Innovation, Faculty of Medicine, Health and Human Sciences, Macquarie University, Sydney, NSW, Australia

**Keywords:** Terminal care, death, end-of-life care, palliative care, bereavement, family, hospital, communication

## Abstract

**Objectives:**

This study explored bereaved relatives’ experiences of end-of-life care (EoL care) in the last 3 days in an acute private hospital in Australia.

**Methods:**

An interpretative qualitative study was conducted. Semi-structured interviews with 8 bereaved relatives whose family member had died at an acute private hospital shared their experiences of the EoL care during the last 3 days of life. The transcribed interviews were analyzed using inductive thematic analysis.

**Results:**

Bereaved family members had mixed experiences, and their primary concerns related to the need for improvements in support for the family; communication; and clinicians partnering with families. The need for family support encompassed care for the person dying and the bereaved relatives, before and during the last days of life, and after death. Bereaved relatives perceived that hospital based EoL care could be positive when the care was collaborative with health professionals, patients, and relatives and there was effective communication.

**Significance of results:**

A patient- and family-centered approach to EoL care should be provided in hospitals, and it requires understanding of the needs of both patients and family members, including informational requirements, communication approaches, and care delivery. Health-care organizations have a responsibility to care for families and this must be considered as part of organizational readiness and ongoing assessment to determine if the standards for EoL care are met. The findings serve as a guide for evidence-informed practice and may contribute to the development of resources and guidelines for delivery of quality EoL care.

## Introduction

Improving and providing quality palliative end-of-life care (EoL care) has become a global priority with the World Health Organization reinforcing the need for palliative care services being integrated into health-care systems (World Health Organisation, 2021). Palliative care encompasses EoL care which is provided to people who are facing the end of their life and to their families (Australian Commission on Safety and Quality in Health Care 2023). EoL care is defined in different ways, from care of people who are likely to die within 12 months to care in the last days and hours of life (Australian Commission on Safety and Quality in Health Care 2023). Most deaths in developed countries occur in hospitals, emphasizing the need for the delivery of quality hospital-based EoL care (Australian Commission on Safety and Quality in Health Care 2023; Australian Institute of Health and Welfare 2019; Gallagher and Krawczyk 2013; National Palliative and End of Life Care Partnership 2021).

Bereaved families’ experiences of hospital-based EoL care of adult patients in North America, Scandinavia, Europe, Australasia, and New Zealand are varied; however, all expressed some concerns about different aspects related to the quality of the EoL care (Ó Coimín et al. 2019; Robertson et al. 2022; Walker et al. 2022). The key concern was poor communication between staff and relatives. This was related to staff communication styles, rushed communication, lack of communication of critical information about dying and preparation for death, and how this led to family distress and dissatisfaction (Caswell et al. 2015; Jackson et al. 2019; Ó Coimín et al. 2019; Robertson et al. 2022; Walker et al. 2022).

Several other areas of concern about EoL care have been reported by bereaved family members (Walker et al. 2022). These include expectations of clinical care, opportunity to participate in patient care, symptom management, need for spiritual support, the environment of care, need of support for patients and families, and the desire to be involved in decision-making (Moon et al. 2024; Ó Coimín et al. 2019; Robertson et al. 2022; Walker et al. 2022; Zhu et al. 2024). Theseconcerns reflect the complexities of providing EoL care including the physical, psychosocial, spiritual, and environmental alongside the expectations of families.

Contrastingly, bereaved family members in New Zealand reported satisfaction with EoL care when case was based upon (1) an empathetic relationship; (2) effective interactions between patients/families and staff; (3) contextualized knowledge of the patient/family; and (4) staff, patients, and families being active participants in care (Gott et al. 2019, 797).

Previous studies have found that the place of death in a hospital, such as a general ward, palliative care unit, or intensive care, may also impact on the quality of EoL care and the patient, family, and staff experiences (Robertson et al. 2022; Rolnick et al. 2020). Dedicated palliative care wards differ from other hospital wards as the staff and volunteers usually have palliative care training and palliative wards have a psychosocial focus in comparison with busy, often noisy, acute wards with staff who may not have had palliative care training (Miller et al. 2023). Intensive care units have been viewed positively by some family members due to better staffing ratios and clinical care (Rolnick et al. 2020).

In Australia, EoL care services are provided at federal, state, and territory levels, in both public and private hospitals and community settings with some government funded services. The services are guided by the EoL care “National Consensus Statement: Essential elements for safe and high-quality end-of-life care” (Australian Commission on Safety and Quality in Health Care 2023), alongside the “National Safety and Quality Health Service Standards” (Australian Commission on Safety and Quality in Health Care 2021), and the “National Palliative Care Standards for All Health Professionals and Aged Care Services” (Palliative Care Australia 2024). Strategies are also in place at state levels, such as the “West Australian End-of-Life and Palliative Care Strategy 2018–2028” (WA Cancer and Palliative Care Network 2018). These guidelines all state the importance of involving patients and families in EoL care.

Recent studies have examined EoL care in Australian acute hospitals (Maubach et al. 2019; Mitchell et al. 2021; Saunders Seaman et al. 2021); however, there are few studies exploring relatives’ experiences of EoL care (Clark et al. 2015; Odgers et al. 2018); and no studies examining bereaved family experiences in Australian acute private hospitals.

In Australia, a private hospital is owned and operated by private for-profit companies or not-for-profit organizations and requires licensing by relevant state or territory governments (The Department of Health and Aged Care 2024). Patients receiving care at a private hospital are required to pay via their private health insurance or through self-funding, and part of the fees may be covered by Medicare, the Australian medical benefits system (The Department of Health and Aged Care 2024). In Australia, there were 697 public hospitals in 2021–22, and 657 private hospitals registered in 2016–17 (most recent data) (Australian Bureau of Statistics 2018), with 2 out of 5 hospitalizations in a private hospital (Healthdirect Australia 2022). Approximately 45% of the Australian population has private hospital health insurance and about 40% of hospital admissions are to private hospitals (Duckett and Nemet 2019). In 2021–22, the majority (56%) of palliative care-related hospitalizations ended with the patient dying in hospital, with 50% in private hospitals, and private health insurance funding 75% of the admissions (Australian Institute of Health and Welfare 2023). There continues to be a need to improve EoL care in all settings including acute private hospitals, and further research of bereaved relatives’ experiences, can inform improvements in care.

## Methods

### Design and setting

This study utilized an interpretative qualitative design to explore bereaved relatives’ experiences of EoL care in an acute private hospital. The episode of EoL care was determined as the last 3 days of life. This study was a component of a multifaceted study examining the quality of EoL care in an acute Australian private hospital that included an observational study (Saunders et al. 2021b) and a mixed-method study of clinical staffs’ perceptions of EoL care (Saunders Glass et al. 2021).

This study was conducted at the largest acute Australian private hospital (with over 800 beds) located a metropolitan area of Western Australia. EoL care is provided, as needed, to patients on all ward 24/7, and the hospital also has dedicated palliative care beds on one ward. The model of palliative and EoL care is guided by the “West Australian End-of-Life and Palliative Care Strategy 2018–2028” (WA Cancer and Palliative Care Network 2018). The hospital has a multidisciplinary palliative care team, led by palliative care consultants in collaboration with nurses, allied health professions (occupational therapists, physiotherapists, dieticians, social workers, and pharmacists), chaplains, and volunteers. The palliative care team supports all patients who are admitted under the palliative consultant, and other patients referred during their hospital admission by other specialists. The hospital is known for their high standard of palliative care and one of the palliative care physicians established the first hospital-based palliative care unit in Australia.

### Participants

A purposive sample of relatives (recorded in the patient’s medical file as “next of kin”) of adult patients who died at the hospital between July and December 2017 were recruited. The inclusion criteria were: (a) family member of a patient during their last 3 days of their life at the hospital; (b) being 18 years old or above; (c) being able to communicate in English. The exclusion criteria were: (a) relatives of patients whose death occurred as a “sudden death” (defined as death within 4 hours of admission); or (b) death under coronial investigation; or (c) death of a patient in the mental health ward.

### Recruitment

A postal recruitment package (participant information form; expression of interest form; and a stamped addressed envelope) was sent out to 148 potential participants at least 6 months post-death of their family members. Our timeframe was guided by research and 6 months was determined as reasonable when making contact following the death of their family member (Bentley and O’Connor 2015). After receiving the expression of interest form, a research team member contacted the interested participant(s) via phone or email to provide further information and arrange a convenient time and location for the interview. There was no follow-up to eligible participants who did not respond to the recruitment invitation.

### Data collection

Participants could elect for the interview to be face-to-face at the hospital or via telephone. Telephone interviews enabled participants who were not living near the hospital or who preferred not to return to the hospital to be interviewed. Informed written consent was obtained prior to each interview. Participants were informed the interview would be recorded, of their right to withdraw from the study at any time without giving an explanation, as well as the option to pause the audio recording or reschedule the interview. Due to the sensitive nature of the topic, participants were provided with information of support organizations to contact should they become distressed.

Semi-structured interviews, with open-ended questions, were conducted from 1 November 2018 to 13 December 2018. The interview guide (Supplementary File 1) consisted of 8 key areas: (1) bereaved relatives’ overall experience of EoL care received; (2) their expectations of EoL care; (3) communication with staff; (4) information received about EoL care; (5) symptom management; (6) support; (7) EoL care concerns; and (8) delivery of EoL care. The interview guide was developed based upon evidence from literature and a review by 2 members of the hospital consumer advisory committee based on their individual experiences and perceptions of EoL care. Interviews were conducted by 3 researchers (RS, JA, AW). As part of the planning for the interviews, the researchers met to discuss and clarify the nature of the questions and identify prompts to limit data collection bias. No time limit was placed on the interview duration, as other EoL care research found participants needed to share their experiences within time frames to meet their individual needs (Bentley and O’Connor 2016).

### Data analysis

Braun and Clarke’s (2013) 6-step framework was utilized for the inductive thematic analysis. Transcripts were transcribed verbatim, uploaded, and analyzed utilizing NVivo 12 (Lumivero 2023), by 3 researchers (CG, RS, KS) who independently read transcripts numerous times to become familiar with the data. The researchers then independently coded the transcripts, then following discussion assembled codes into initial themes before collating them into final themes. All themes were then reviewed to ensure they were a true representation of the data. The review of themes was completed by all researchers collaboratively to enhance the rigor of the research (Nowell et al. 2017).

## Results

Eight interviews were conducted (five face-to-face and three telephone) with seven female and one male relative (including three spouses, three offspring, and one in-law) of patients who received EoL care in the hospital. One patient died in ICU, one on a general ward, and others on the palliative care ward. One interview had two bereaved relatives present, with the rest being interviewed individually. Interviews were 45–90 minutes in duration.

### Family experiences

Families’ experiences of EoL care was described in 3 themes: (1) *Support for families*; (2) *Communicating with families during* EoL care; and (3) *Partnering with families during* EoL care.

#### Theme 1. Support for families

The bereaved relatives described their “support needs,” which related to both the support they received and expected from the hospital. The different care approaches, relating to the health professional interaction with family members, impacted their experiences. Family members who had positive experiences explained that their perception of the care provided enabled a sense of safety and confidence. Further, participants appreciated the respect they received and the opportunities to be involved in the care of their loved one.

They treated me with tremendous respect and involved me in all the discussions that took place. (Participant 1)

In contrast, some shared being left alone with their family member who was dying and would have preferred more interaction, reassurance, and support from staff. A few participants stated that they would have appreciated having someone to sit with them and provide some physical contact such as holding their hand.

It was like she was just left there, we were left […] I can’t remember if anyone was there when we had like a family meeting. (Participant 6)

Participants shared that informational, emotional, and spiritual support were seen as helpful, as the hospital’s spiritual service(s) supported both themselves and patients.

Informing us of our, what we could do to be with him … they would just come and talk to us about dad’s life and about our family … I think that was a good emotional outlet for us to be able to remember all the times with dad. (Participant 3)

Emotional support, after the person died, was also important for family members. Some described experiencing stress as they “grappled” with their reactions immediately after the death, prior to leaving the hospital, when support was not adequately provided.

… they gave us all the stuff, the forms we had to fill in … but then they just left us … and then we packed up and then as we, actually it was, as we walked past the nurse’s station, they just said see you later … I think one of them said sorry for your loss or something briefly as we walk past, but they never come and offered us a cup of tea. (Participant 8)

One family member also found the immediacy of the request for information about funeral directors after the family member died was considered stressful and insensitive.

The thing that really upset me is that as I walked out, they said, “well, what funeral directors are you using, … and advised that’s what you need, … these are the things that you’ve got to organise” … I knew it was going to come, [and] we should have done it, but hadn’t even thought that I had to do it right on the spot sort of thing. (Participant 7)

The environment where the EoL care was provided was seen to be important to support family members and the patients. The busy acute wards were recognized as not being able to meet EoL care needs and access to private areas and the outdoors was seen as important for family to spend time with the patient who required EoL care.

There wasn’t really anywhere nice to go. … who wants to live in a hospital room for the last week of their life sort of thing. I wanted to get her out in the sunshine and the fresh air and everything … I found a little garden but to get there I had to push her down this really steep ramp, or two ramps and then to push it back up again and when I had the wheelchair with the oxygen it was so heavy. (Participant 6)

#### Theme 2. Communicating with families during EoL care

The importance of communication for bereaved relatives was seen as critical and effective communication supported the family when communication was clear, it helped reassure the family that the care given to their loved one met their expectations.

We were confident that dad was being cared for when we were not there … We had a completely open conversation about everything including his expectancy in terms of dying, how we should deal with that, how he was feeling. It was an extraordinarily strong bond that we developed, and I felt always that, they were just really concerned about making sure the family was happy with what was happening. (Participant 1)

In contrast, experiences of ineffective communication impacted on the family experiences and where there was a lack of information and clear communication it increased the stress levels of family members. Communication with family members was identified as particularly important with patients who were not able to understand the information due to their illness or other disease processes.

… We would have liked more direct communication. Because Dad had dementia too, we didn’t, like he couldn’t give us feedback if the doctor had been. (Participant 3)

Some participants experienced ambiguity and confusion regarding patient care, due to doctors communicating at different points and providing varied information.

… The doctor would come and see her at different times and talk to us at different times and we’d be getting conflicting information, conflicting ideas… (Participant 6)

Additionally, family members were inadequately prepared for the symptoms that can occur at the end of life.

I felt like yeah it was a shock to, to with the death rattle that came with it. (Participant 5)

Non-verbal communication by staff including eye contact, touch, being present, and body language was recognized as an important part of care. One participant questioned if care approaches were linked to the staff personal culture.

They were all very polite, very, you know very efficient all of those things but there was no, I think it’s a cultural thing, you know there’s no touch, there’s no you know holding my hand … When xx passed away there was a xxx man and an xxx woman, I was on my own again and there was absolutely no connection there again. Again, all very efficient and polite but I needed someone just to sit with me for 5 minutes and hold my hand. (Participant 5)

#### Theme 3. Partnering with families during EoL care

The involvement of families was identified as being essential for safe and quality EoL care. Although the clinical abilities of the health-care professionals were valued, clinician’s ability to collaborate and work together with the patient and families was seen as equally critical.

She [doctor] and I sat with him, and we had a really honest conversation with him about that we were working together, and we were trying to help, and we were responding to him, so, you know, if he asked us for help between us we would deal with it … we were both working together as a team and I thought that was wonderful. (Participant 1)

Family members wanted to be involved in the care and for the staff to understand the family perspectives of care to assist in determining the most appropriate and effective plan of care. Key to understanding the family’s perspectives was listening to the family about their observations of the patient during the dying process.

… They just listened you know and just made the whole experience a little bit easier I think for us to manage. (Participant 3)

Negative experiences left family members feeling “traumatized,” particularly when they were not listened to about the patient’s pain management needs and subsequently observing the patient experiencing severe pain. Family members expected clear information about the reality of the situation but were unaware that staff would not discuss the patient’s condition without the patient’s consent.

I now see him deteriorating each day, I need to know [if] I need to have my children come down here and you [the doctor] need to be honest with me about his prognosis and what’s going on. [And the doctor said:] ‘that is none of your business, that is patient confidentiality, and I will not be saying one word to you about this patient. If you want to know what’s going on with your husband, you ask your husband’. I And I said you expect me to … ask my husband, [that] you are not going to make it [and] you are going to die? (Participant 2)

## Discussion

Families’ experiences of hospital based EoL care were complex and overlapped with their individual perceptions, needs, and expectations. The overarching finding of this study was the need for support. In the participants’ experiences, support for families meant understanding and accommodating family’s expectation with care delivered, their support needs, and maintaining open communication between family members and health professionals. Here the concept of “support for family” was often interchangeable and overlapped to include support for the bereaved families and/or support for the patient receiving the EoL care. Families had an expectation of quality EoL care for the person dying, and this influenced bereaved relatives’ experiences and their perception of dying in hospital. Relatives expected the EoL care to make the dying “comfortable” and when this occurred it made the relatives comfortable with the care, but in contrast inadequate care left the family member distressed (Ó Coimín et al. 2019; Robertson et al. 2022).

Other findings have also highlighted that a clear understanding, information about approaching EoL care, and how the decision of palliative care was made affected participants’ experiences. These findings are not unique to the private hospital as they resonated with other recent studies that found supporting individual needs was the hallmark of bereaved relatives’ satisfaction of care (Walker et al. 2022) and that communication and the humanness of health-care professionals were more impactful then the setting of the care given (Robertson et al. 2022)

A family’s expectation of clear information and communication regarding the patient at the end of life, and how this affected bereaved relatives’ experiences, has been reported over many years (Bussmann et al. 2015; Johnson et al. 2021; Ó Coimín et al. 2019; Robertson et al. 2022; Walker et al. 2022; Witkamp et al. 2015). Our study reinforces that communication with families still requires improvement and effective communication can contribute to a more supported experience. Factors associated with positive experiences included adequate and supportive communication, staff spending adequate time with the patient and family providing emotional support and education, and involving patient and family in care decisions (Bussmann et al. 2015; Donnelly and Psirides 2015; Gallagher and Krawczyk 2013; Gott et al. 2019; Noome et al. 2016; Ó Coimín et al. 2019; Robertson et al. 2022; Stajduhar et al. 2017; Walker et al. 2022).

The partnership between patients, families, nursing staff, and other health-care professionals was viewed positively by most family members. Good clinical skills and effective therapeutic communication, including non-verbal skills and the use of touch, increased levels of satisfaction. Health-care organizations need to ensure that adequate requirements are in place for EoL care including staff resources, education and staff training, and appropriate environmental areas for care (Ó Coimín et al. 2019; Rawlings et al. 2022; Robertson et al. 2022; Saarinen et al. 2023). For people at the end of life and their families, an environment that provides space for family interaction and privacy is important (Robinson et al. 2018). In this study, the hospital environment presented a challenge for family members finding private spaces and accessing the outdoors with the patient. Both of these can led to increased levels of stress, anxiety, and insecurity, which is often a limitation of acute hospital environments for patients and families (Brereton et al. 2012; Johnson et al. 2021; Robertson et al. 2022). Other studies have recommended that patients, nearing their end of life, should have dedicated individual rooms to facilitate privacy to enable a better environment for families to spend the last days of life with the patient and be able to have sensitive discussions with clinical staff (Kim et al. 2023; Ó Coimín et al. 2019; Otani et al. 2022).

The findings highlight the bereaved family members were expecting respectful care involving family, effective communication, and excellence in clinical care; however, their experiences are linked to challenges of providing EoL care in acute hospitals. Hospitals should be able to provide quality EoL care, however due to the acute nature of the hospital clinical environment and lack of competent staff, compared with dedicated palliative care environments, quality EoL care is not always achieved (Rawlings et al. 2022).

Our findings have important implications for improvements in practice, policies, information for families, and further research. As an outcome of this study, several practice changes were implemented, including the introduction of a screening tool on admission to identify if patients have palliative needs, development of new educational resources for families, and staff education.

### Limitations

Although this study has enabled an exploration of bereaved relatives’ experiences of EoL care in an acute private hospital, there are limitations. The voluntary participation may indicate a possible self-selection bias, and the recruitment of participants 6 months post-death to participate may have impacted participant recall. Findings are limited to experiences in a private Western Australian hospital and an English-speaking participant population which hinders the generalizability of the results. Experience of care may differ in bereaved relatives from culturally and linguistically diverse backgrounds. Thus, future research is needed to explore the experiences of EoL care for people from diverse backgrounds.

## Conclusion

Bereaved family members had mixed experiences and the competing demands of an acute hospital environment may have contributed to the challenges they experienced. Interviewing bereaved family members about their EoL care experiences has reinforced the importance of respect, presence, trust, and communication in providing care to patients and families. Health professionals need to strive to provide consistent safe and high-quality EoL care to patients irrespective of the setting, and health-care organizations have a responsibility for policy and EoL care practices including care of family. Failure to achieve this may result in a long-lasting negative impact.

## Supporting information

Saunders et al. supplementary material 1 Saunders et al. supplementary material

Saunders et al. supplementary material 2 Saunders et al. supplementary material

Saunders et al. supplementary material 3 Saunders et al. supplementary material

## Supplementary material

The supplementary material for this article can be found at https://doi.org/10.1017/S1478951525000045.
