# Supplementary material for: Families’ experiences of end-of-life care in an acute private hospital: A qualitative study
Source: Palliat Support Care. 2025 Feb 28;23:e72. doi: 10.1017/S1478951525000045 (PMC13166413; doi:10.1017/S1478951525000045)
Supplement: Saunders et al. supplementary material 1 — Saunders et al. supplementary material [file S1478951525000045sup001.docx]

*Acknowledgements*

We thank Hollywood Private Hospital for fostering collaboration allowing research to improve quality of care provided, and consumer representatives that provided feedback on tool development. Thank you to research assistants Michelle Gay and Paige Watkins. Thank you especially to all bereaved family members who participated in the study.
