# Supplementary material for: Families’ experiences of end-of-life care in an acute private hospital: A qualitative study
Source: Palliat Support Care. 2025 Feb 28;23:e72. doi: 10.1017/S1478951525000045 (PMC13166413; doi:10.1017/S1478951525000045)
Supplement: Saunders et al. supplementary material 2 — Saunders et al. supplementary material [file S1478951525000045sup002.docx]

**Families’ experiences of end-of-life care in an acute private hospital: A qualitative study.**

**Interview Guide**

1. Reflecting on the end-of-life care provided to xxx during their last three days of life, can you please share your experience of the end-of-life care that was provided?
2. Overall, how well informed were you about xxx’s condition over the course of his/her hospitalisation?
3. Did the health care team discuss what to expect physically and emotionally about xxx who was dying?
4. How did the health care team discuss the end-of-life care plan with xxx, you and your family?
5. During the last 3 days of care how was xxx, you and your family involved in decisions about xxx’s care?
6. Can you tell me about how the health care team managed xxx’s symptoms, in the last 3 days of care?
7. Can you tell me more about how the health care team supported you and/or your family during xxx’s last 3 days of care?
8. From your experience, was there any concerning aspect of the end-of-life care that was provided to xxx during their last three days of life?
